# Pathways to Care: Duration of Untreated Psychosis from Karachi, Pakistan

**DOI:** 10.1371/journal.pone.0007409

**Published:** 2009-10-12

**Authors:** Haider A. Naqvi, Sajjad Hussain, Muhammad Zaman, Mohammad Islam

**Affiliations:** 1 Department of Psychiatry, Aga Khan University, Karachi, Pakistan; 2 Department of Community Health Sciences, Aga Khan University, Karachi, Pakistan; University of Muenster, Germany

## Abstract

**Background:**

Substantial amount of time is lost before initiation of treatment in Schizophrenia. The delay in treatment is labelled as Duration of Untreated Psychosis (DUP). Most of these estimates come from western countries, where health systems are relatively better developed. There is dearth of information on pathway to care from developing countries.

**Methods and Results:**

Patients with ICD-10 based diagnosis of Schizophrenia were enrolled by convenient method of sampling. The pathway to care was explored through a semi-structured questionnaire. Onset, course and symptoms of psychosis were assessed using Interview for the Retrospective Assessment of the Onset of Schizophrenia (IRAOS). Ethical approval of the project was taken from The Aga Khan University, Ethics Review Committee. Of the enrolled 93 subjects, 55 (59%) were males and 38 (41%) were females. In our sample, 1.56 mean (median, 2) attempts were made prior to successful help seeking. The duration of untreated psychosis was 14.8 months (St. Deviation; 29.4). DUP was 16.8 months (St. Deviation; 34.9) for males and 11.8 months (St. Deviation; 18.9) for females. In the pathway to care, psychiatrists featured prominently as initial care providers. In the first attempt at help-seeking, 43% patients were initially taken to psychiatrists. After the initial consultation, 45% were prescribed psychotropic medication while 7% were hospitalized. Only 9% subjects were given the diagnosis of schizophrenia initially. When participants were inquired about the reasons for delay, 29% reported financial difficulties as the barrier to care. Positive symptoms of psychosis were present in 57% subjects while negative symptoms were present in 30% subjects. There was a statistically significant difference (Chi-square; 7.928, df: 1, Sig 0.005) between DUP and the positive and negative symptoms category.

**Conclusion:**

In the absence of well developed primary care health system in Pakistan, majority of patients present to psychiatrists as a first contact. DUP, as a measurement of help seeking behaviour, tends to be shorter with positive symptoms of Schizophrenia. Substantial amount of time is lost due to non recognition of disease and subsequently, inadequate treatment. Secondary prevention strategies should focus on families, which play an important role in the treatment-seeking process of psychotic patients.

## Introduction

Estimates of time between onset of psychosis and initiation of treatment – duration of untreated psychosis - is estimated to be 22 weeks to over 150 weeks [1]. There are variable predictors of treatment delay in Psychosis. According to some studies, positive symptoms of psychosis (hostility) predicted short delay while negative symptoms predicted longer delays [2]. However other studies fail to confirm this association [3]. Social participation (social network outside family, employment) predicted shorter delay according to some studies [4], [5]. In a study by Okazaki, exploring delay in Americans of Asian origin, low educational level and perceived stigma were associated with significant delay in help seeking [6]. Other factors like substance use, homelessness and contact with law, propensity to seek help, service accessibility and stigma have also been reported [7]–[9].

General practitioners (GP) are considered to the gate keepers to specialized psychiatric services. This is somewhat similar to the seminal work of Goldberg & Huxley which proposes that patients have to pass through different filters in order to reach specialized mental health care [10]. If we examine the literature on pathway to psychiatric care, there is a wide variation in referral rate by general practitioners. There are factors related to general practitioners (GP) and the patients [11]–[13]. In an epidemiological catchment area study, people who developed mental illness during follow-up interval consulted non-psychiatrist physicians as often as specialists. There was no difference between affective disorders and psychotic disorders [14]. In a comprehensive study of pathway to psychiatric care, looking at sixty two first-episode of psychosis in Australia, Lincolin et al. reported a mean treatment delay of 16 weeks [15]. As some individuals received ineffective help in the community, the number of weeks between first symptoms and treatment was 58 weeks. Their findings indicated that family practitioners potentially have a key role in identifying the signs and symptoms of mental illness, since many of the initial help seeking attempts were addressed by family doctors. Norman et al. (2004) described a care pathway of 110 first-episode patients with Schizophrenia in an early intervention program for psychosis in London, Canada [16]. Family physicians and hospital emergency rooms were prominent components of pathways to care. Although there is some evidence that pathway taken by patients in Pakistan is through community and general practitioner before arriving to specialized mental health services , other pathways like referral from native faith healers are also used more frequently [17].

There is limited data on treatment delay in psychosis from developing countries. A study from Ghana, reports a median delay of 6 months from onset of symptoms to treatment contact [18]. A pilot study from Aga Khan University Hospital looked at the generic pathway to psychiatric care. There appeared to be significant delay between the onset of first symptoms and presenting to the first health carers' with a mean delay of 2.8 years [19]. There is no data on DUP and pathway to care in Schizophrenia from Pakistan, a South East Asian developing country. This is despite the fact that studies have reported a better prognosis of schizophrenia from developing countries.

## Materials and Methods

AKUH is a 500 bedded private teaching hospital, located centrally in Karachi (population approx. 16 million), Pakistan's largest city and its main business and commercial centre. The hospital provides service for almost all specialties including psychiatry. It has a 24-hour emergency room. Since its inception almost 25 years ago, the hospital has gained a reputation for high quality medical care in the region and attracts patients from all over the country. Although there is no referral system in operation, the hospital is viewed as a tertiary referral centre and regularly receives some of the most complex medical and psychiatric cases.

The department of psychiatry at AKUH comprises of five full time and three part time psychiatrists as well as three clinical psychologists. There is an eighteen bedded in-patient unit. Outpatient services consist of 44 clinics per week including a specialist child Psychiatry clinic. There is a provision of low-cost Community Health Clinics for those patients who cannot afford to pay. In case, when patients cannot afford to pay at all, AKUH, welfare department would generally cover for all incurred charges. Medical records are kept in a seven digit confidential file, maintained by Health Information and Management System (HIMS). A separate file is maintained by the department of Psychiatry, considering the issue of confidentiality and privacy.

Ethical approval: Ethics review committee (ERC) of Aga Khan University Hospital reviewed the study protocol and granted permission to conduct it. Study was conducted in compliance with ethical principles for medical research involving human subjects' of the Helsinki Declaration.

For purpose of enrolment, the study protocol was also presented in the Department of Psychiatry, AKUH, with a specific request made to individual faculty members for patient-referrals. Patients and their families were subsequently briefed about the project. It was emphasized that there would be no loss of benefit, or compromise in long term care, in case of non-participation. Written informed consent was taken from all participants. For the sake of confidentially no names or identifiable information was recorded.

Study participants: In the initial intake interview patients were seen independently by a consultant psychiatrist. A detailed history and examination was carried out as a routine. Medical record requires that patients are given a definitive or probable diagnosis; based on the assessment, patient's care plan is subsequently planed. Medical records are reviewed periodically and subsequently definitive diagnosis is generated. Patient with ambiguous diagnoses are discussed in patient care meetings, held weekly, attended by all faculty members. The final diagnosis was used for patients' enrolment.

All patients with an ICD-10 diagnosis of Schizophrenia, seeking help at ambulatory care clinics, were enrolled consecutively for the study. Patients admitted in the psychiatry ward were also approached for participation. A centre based, convenient method of enrolment was followed. Patients with schizoaffective disorder or co-morbid substance abuse, mental retardation and organic mental disorders were excluded from the study. A screening MRI Brain was carried out, as part of the project, in order to exclude any physical abnormality.

Data collection: In a separate interview a research medical officer, hired specifically for the project, collected the research data. . He was trained by the P.I (HN). Each case was reviewed with the P.I. (HN) and if consensus was not reached on the final diagnosis then further information was sought from the primary psychiatrist. Only patients with definitive diagnosis of schizophrenia were enrolled.

### Questionnaires

#### Pathway to Care

The pathway to care was explored through a questionnaire which explores the help seeking behavior since the onset of symptoms. The questionnaire was originally developed by Perkins, Nierie and Bell, from University of North Carolina, Chapel Hill, North Carolina. It was adapted for local use.

The questionnaire specifically inquired about

- When did they first feel that they needed help?
- Who realized the initial change in behaviour - symptoms of the illness?
- What/when was it noticed?
- How many times did they try before receiving any treatment?
- What was it that made them seek help? What were the initial symptoms?
- Who was contacted for initial symptoms of the illness/change in behaviour?
- What was done?
- What advice was given? What was told about the problem?
- How helpful was the advice?
- What were the reasons for delay in help seeking/What were the barriers to care?

#### Interview for the Retrospective Assessment of the Onset of Schizophrenia (IRAOS)

In order to measure the onset and course of psychosis ‘Interview for the Retrospective Assessment of the Onset of Schizophrenia (IRAOS). IROAS was originally developed within the framework of the ABC (Mnemonic for Age, Beginning and Course) schizophrenia study (Häfner et al, 1998) for systematic research on the onset of schizophrenia [20]. It is one of the most robust, widely used instruments in the Schizophrenia-research. IRAOS examines the social course, course of the symptoms, disability and treatment from the first signs or indicators of illness until the time of interview.

In the socio-demographic part of the instrument the entire life of the patient is thoroughly recorded. With the help of ‘continuity item’, based on six key social roles, breaks in social development and their causes are assessed. The part on ‘indicator’ deals with a retrospective recording of symptoms that might be “indicator” of a beginning psychiatric illness or are relevant to diagnosing the disorder in question. IRAOS queries psychotic and non-psychotic symptoms against the time line called calendar of episode. This not only helps in detecting the particular type of symptoms, but also shows the longitudinal course of particular symptoms. Urdu versions of both the instruments were used in this study.

### Measures

#### Onset of Psychosis

Onset of psychosis is delineated by nonspecific symptoms or ideographically unusual behavior at the start of prodromal phase. Then there is the appearance of first psychotic symptoms. Afterwards there is presentation for treatment. First, questions were asked about the first signs and symptoms. The appearance of new symptoms and the change in severity of such symptoms was documented. These were noted in chronological order.

#### Duration of untreated psychosis

Duration of untreated psychosis was determined by assessing the age when the first psychotic symptom (symptoms must have lasted throughout the day for several days or several times a week, not being limited to few brief moments) were present and then the age at which first effective treatment was initiated. For the purpose this study an adequate course of treatment was defined as at least 6 weeks of treatment with haloperidol 5 mg or equivalent daily dose.

#### Concerns

By eliciting symptoms and problems this helped subjects and their families do define the concerns or problems for which they sought help. We defined a concern as a distinct behavior that was (i) deviant from the usual pattern of behavior, (ii) clearly remembered, (iii) made them think that something was wrong, and (iv) seemed to be related to onset.

#### Pathway to care

Subjects and family members were asked about all services sought by family and subjects to address the onset of symptoms. Services included all professional (mental health, primary care physicians, etc) and non-professional (e.g. friends) avenues.

The number of help seeking contacts is defined as the number of individuals sought out for assistance for mental health concerns. All help seeking attempts prior to, but not including the contact, that offered the antipsychotic medication treatment and proposed appropriate treatment for psychosis are referred to as unsuccessful help seeking attempt. A contact, that resulted in what has been defined as an adequate course of medication is defined as successful (as defined earlier, haloperidol 5 mg or equivalent for the duration of six weeks).

### Data Analysis

Data was initially entered in the Epidata. This software allows the flexibility of creating multiple fields, as relevant to handling large data sets. Files from Epidata were subsequently transferred to Statistical Package for Social Sciences 15.0 (SPSS, Inc., Chicago, Il, USA) for further analysis. Variables pertaining to IRAOS were merged with the pathway to care data. This enabled us to check the internal consistency on many variables. For example age at onset, duration of untreated psychosis, constellation of symptoms and earliest symptoms/behavioral concerns, etc).

DUP was taken as an outcome variable. It was dichotomized into early and late, based on 6 months duration. Previous literature supports this statistical measure. Chi-square test of association was applied for dichotomous variables. Univariate analysis was carried out on socio-demographic (gender, marital status, education level), family history of psychiatric illness and concerning behaviour. For continuous variables, Student's T test was used, significance level, 0.05.

## Results

Socio-demographic characteristics: This study enrolled 93 subjects; 55 (59%) males and 38 (41%) females. The mean age for males was 35 years (St. Deviation 10.4) while for females it was 32 years (St. Deviation 10.0). The number of married males (44%) was greater than married females (30%). There was no significant difference in the mean age at onset of illness. The mean age at onset, as calculated from the onset of first signs of illness, was 23.54 years in males (St. Deviation 7.3; median 24 years) while 23.92 years in females (St. Deviation 9.34; median 24 years). Table-1 describes the gender-specified socio-demographic variables.

**Table 1:** Socio-demographic characteristics of study participants.

	Male (n)	%	Female (n)	%
Gender	55	59	38	41
Marital Status				
Single	15	39.5	32	58.2
Married	17	44.7	17	30.9
Divorced	6	15.8	6	10.9
Religion				
Muslim	42	76.4	34	89.5
Christian	1	1.8	1	2.6
Others	12	21.8	3	7.6
Education				
Illiterate	5	9.1	7	18.4
Primary	1	1.8	0	0
Secondary	12	20.8	7	18.4
Intermediate	35	63.6	23	60.5
Bachelors	2	3.6	1	2.6
Continuity of education				
Completion in normal time	36	65.5	22	57.9
Completion with some delay	11	20	7	18.4
Change in school	1	1.8	2	5.3
No information	7	12.7	7	18.4
Vocational training				
None	26	47.3	21	57.9
Technical college training	2	3.6	0	0
Polytechnic training	1	1.8	0	0
University	24	43.6	16	42.1
Not relevant	2	3.6	1	2.6
Main Occupation				
Office work	9	16.5	2	4.6
Business	6	11	1	2.6
Employed	10	19.5	3	7.9
Administrative/academic	12	22	0	0
Housewife	0	0	20	52.6
Student	6	11	2	5.3
Unemployed	11	20	10	26.3
Current Occupation				
No work	32	58.2	16	42.1
Regular work, in normal setting	20	36.4	0	0
Industrial therapy	1	1.8	0	0
Other	2	3.6	1	2.6

Treatment attempts: In our sample, 1.56 (mean; median, 2) attempts were made prior to successful help seeking.

Duration of untreated psychosis: The mean duration of untreated psychosis was 64 weeks (Months; 14.8, SD; 29.4). Mean DUP was 16.8 months (SD: 34.9) for males and 11.8 months (SD: 18.9) for females. There was no significant association between DUP and Gender (Chi-square = 0.003; df = 1), marital status (Chi-square = 1.157; df = 2), education (Chi-square = 0.05; df = 2), and continuity of school education as a measure of pre-morbid functioning (Chi-square = 0.329; df = 2).

### Pathway to care

In the pathway to care, psychiatrists were most commonly approached as the initial care provider. In the first episode of psychosis, 43% patients were initially taken to the psychiatrist. This was followed by faith healers, who were visited by 15% of the patients. Around 5 % of the participants visited general practitioners and the emergency department each, while rest visited teachers, social workers, psychologists and other caregivers for help.

**Figure 1:**
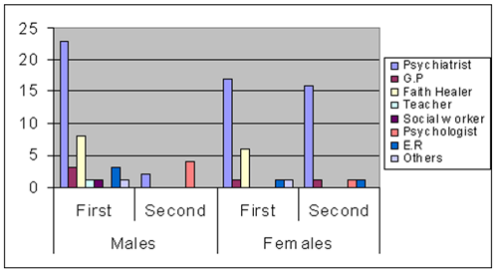
Care providers stratified by gender and number of help seeking attempts (n).

If the first attempt was unsuccessful, i.e. appropriate treatment was not initiated; patients were asked about the second attempt at help seeking. In the second attempt for treatment, 47% of the participants visited psychiatrists, while a small minority (4%) consulted clinical psychologists. Figure-1 describes the care providers stratified by gender and help seeking attempts.

In 41% of participants', the family noticed the initial change in behavior or onset of symptoms, while 11% subjects themselves noticed the change.

The main reasons for seeking treatment were psychiatric symptoms (35%) and early symptoms of illness (10%). Patient's safety and safety of others were reported by 16% and 2% of the participants, respectively. Similar trend was followed in the second attempt to seek help with the exception of safety of others, which was more of a concern. However, this difference was not statistically significant. Auditory hallucinations (61%), delusions of persecution (43%), irritability (43%), insomnia (41%) and mistrust/withdrawal (29%) were the main concerning behavior which initiated the help seeking behavior. Table-2 describes the concerning behavior at the time of presentation.

**Table 2:** Concerning behaviour at the time of contact.

Concerning Behaviours	Response		Percent of Cases
	n	Percent	
Auditory hallucinations	57	12%	61.3%
Delusions of persecution	40	8.4%	43.0%
Sleeplessness	40	8.4%	43.0%
Irritability	39	8.2%	41.9%
Withdrawal/mistrust	27	5.7%	29.0%
Anxiety symptoms	33	6.9%	35.5%
Other hallucinations	21	4.4%	22.6%
Thoughts broadcast/insertion	22	4.7%	23.7%
Obsessions	23	4.9%	24.8%
Other delusions	22	4.6%	23.7%
Decreased talkativeness/ poverty of speech	16	3.4%	17.2%
Depressed mood	15	3.1%	16.1%
Others	51	10.9	55%

Positive symptoms of psychosis were present in 57% subjects, while negative symptoms were seen in 30% subjects. There was a statistically significant difference (Chi-square; 7.928, df: 1, Sig 0.005) between DUP and the positive and negative symptoms category. This implies that patients with positive symptoms had a shorter DUP while those with negative symptoms had a longer one. Table-3describes the DUP stratified by six months and positive and negative symptoms category.

**Table 3:** Duration of Untreated Psychosis (DUP) and Symptoms category, n = 80.

	DUP (In Months)< = 6 Months	DUP (In Months)> = 6 Months
Positive symptoms: n (%)	37 (78.7)	16 (48.5)
Negative symptoms: n (%)	10 (21.3%)	17 (51.5%)
Total: n (%)	47 (100%)	33 (100%)

In the first episode of help seeking, 46% received no formal treatment while equal numbers (45%) were given psychotropic-medications. Among them, 51% patients did not take the prescribed medications. A small minority (6%) was hospitalized.

When inquired about what they have been told about the diagnosis, 68% reported that nothing had been relayed regarding the diagnosis, while 17% reported that they were told that the patient had some psychiatric illness. Only 8% of the participants' were given the diagnosis of Schizophrenia, initially, while 4% reported that they were told that their diagnosis was Depression. There could be various reasons behind this observation. One could be that patients were assessed in the early phase, when illness was unfolding, with non-specific prodromal symptoms and health care providers were not sure of the definitive diagnosis. Other reason could be quality of consultation. One can only speculate about the quality of communication between health care providers, patients and their families. Interestingly, 44% participants reported that consultation was not helpful at all, while 21% subjects felt that it was a little helpful. Equal number of participants reported that it was ‘very’ helpful.

### Barriers to care

When participants were inquired about the reasons for delay, around 29% reported financial difficulties, whereas 16% cited difficulty in reaching treatment centres. Almost 25% of the patients and an equal number of family members reported that they felt shy in discussing their illness.

**Figure 2:**
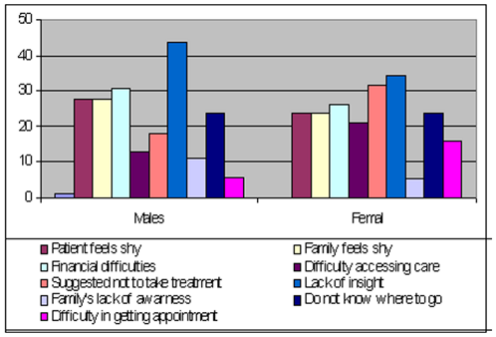
Barriers to care (% of responses).

There were 40% patients' and 9% of family, friends or significant others who did not feel the need for treatment or any urgency to seek help. In around 23% cases, the patients and family did not have information regarding centres that offer treatment. In cases where they knew where to go, around 9% reported difficulty in getting an appointment. Around 5% of the cases reported that health care providers did not take the illness seriously. (See figure 2 for gender stratified data)

## Discussion

This is the first study from Pakistan which examines the delay in help seeking and subsequent duration of untreated psychosis (DUP). To the best of our knowledge there are no estimates on DUP from Pakistan. Our study reports mean DUP of approximately 15 months. This is consistent with findings from other centres [21]. In our study we found no significant gender difference in the age at onset of schizophrenia. An earlier study from our centre has also reported this somewhat discrepant finding [22]. Another important finding is that psychiatrists were the first care providers for 43% of patients. In the absence of well developed primary care systems, most patients present to specialists' by-passing the traditional tiers of care.

### Pathways to care

One can argue that if psychiatrists are the first care provider then DUP should have been less. Though psychiatrists are often seen initially, only a small minority of patients receive the adequate information in time, as apparent from our data. Only 7% of the subjects reported that they were given the diagnosis of schizophrenia, leave alone the other information regarding the disease process, compliance and long term outcome. Not surprisingly, satisfaction levels with the initial consultation were quite low. The economic undertone to the doctor-patient relationship cannot be overlooked, in case of private health care sector. In the scenario of few qualified health care providers' patients throng to these individuals and centres of service. Naturally quality of consultation is compromised.

In this pathway to care study in psychosis more subjects (15%) present to faith healers, then our earlier study on pathway to care for general psychiatric problems (5%). One can also speculate about the reasons behind this finding. Culturally prevalent ideas like black magic, evil eye, etc., as a causative factor, might be the reason for initial help seeking with faith healers.

Another important finding in our study is role of family in help seeking. Psychosis is a condition in which patients have limited insight. Family has an important role in initiating initial care. In almost half of the cases family members noticed the initial change in behaviour. They were instrumental in seeking care, as opposed to other resource persons like friends, teachers, social workers or police. Literature from western countries generally supports this observation. However the role of other individuals should also be recognized. A study by Olin and Mednick (1996) underlines how competent teachers can identify pupils at risk for becoming psychotic [23]. Similarly, early intervention programs have been designed to train general practitioners to identify the early symptoms of the illness.

### Symptoms and help seeking

With respect to help seeking behaviour, patients with positive symptoms had shorter DUP then those with predominant negative symptoms. This difference was statistically significant. Positive symptoms of psychosis might lead to socially disruptive behaviour leading to early help seeking. Similarly a study by Larsen et al. found that patients with negative symptoms and/or a decline in social functioning from childhood to late adolescence have long DUP [24]. However certain positive symptoms like mistrust and persecutory delusions could lead to more resistive behaviour, thereby contributing to delay in help seeking.

Based on results of this study, we feel that early recognition should focus on identifying the prodromal symptoms, insidiously developing negative symptoms and changes in social functioning.

Another way, in which our study is consistent with previous work, is predominance of affective, non-psychotic symptoms in the prodromal phase of the illness. In almost 86% cases, these symptoms were the primary reason for help seeking [25]. This could have contributed to the low recognition rate of psychosis and subsequent prescription of antipsychotic medications. Schizophrenia is also a diagnosis which carries a poor prognosis, requiring life-long treatment. Often psychiatrists are reluctant to label the patients with this condition resulting in prolongation of DUP.

### Socio-demographic characteristics and DUP

In this study there was no significant association of socio-demographic factors (like gender, marital status, education, family history of psychiatric illness) and DUP. In our study since none of the socio-demographic variables were significant at the univariate analysis we could not build a logistic regression model. Though this is consistent with most of the previous studies in help seeking, we feel these quantitative variables might not be able to capture the true determinants. Social interactions provide the basis for social networks, which determine choices and decision making. Utilization research in medical sociology needs an explanatory model, keeping in view the complexities of the disorder itself i.e. psychosis and it's social complications [26].

### Health-services and DUP

We feel that health service related factors might also have an influence on DUP. A median of two attempts were made before prescription of adequate dose of antipsychotic medications. Additionally, critical time was lost due to non recognition of early psychotic symptoms

Almost one third of the sample reported financial difficulties as a barrier to help seeking. In terms of economics, health is considered to be a public good, where government is mandated to provide services. In Pakistan, a south East Asian developing country, the health budget is about 1–2% of the annual Gross Domestic Product (GDP). There is no separate budget for mental health. This makes health care, more or less, an out of pocket expenditure for majority of the general public. There are three main mental health service providers in Pakistan; the specialists in private sectors, the government and traditional healers. Private medical practice in Pakistan is not without problems. It is, in most sections, unregulated and monetary exploitation and abuse of patients in not uncommon. This is particularly so in patients with schizophrenia, due to their variable presenting symptomatology and need for care, which leads to delay, thereby increasing the psychosocial morbidity and illness burden. This is further compounded by the extreme dearth of psychiatrists and other mental health professionals in the country.

Secondary prevention of schizophrenia could be improved by focused and targeted educational interventions involving multiply-affected families and relevant care providers. Traditional as well as alternative care providers might be relevant target groups.

There are certain limitations which should be kept in mind while interpreting the evidence on DUP and treatment seeking behaviour in schizophrenia. Any inquiry on the prodromal phase of illness is bound to have recall bias. Family members of patients with long standing illness, insidious onset and/or negative symptoms also tend to selectively recall the details of early help seeking.

Additionally methodological limitations should also be noted. This was a cross section survey enrolling the patients presenting to a tertiary care hospital, making it not a true representation of the national sample.

In conclusion, help seeking in schizophrenia is influenced by factors related to patients and health services. DUP, as a measurement of help seeking behaviour, tends to be shorter with positive symptoms of schizophrenia. In the absence of well developed primary care health system, majority of patients and families present to psychiatrists as a first contact. Substantial amount of time is lost due to non recognition of prodromal symptoms and inadequate treatment. Secondary prevention strategies should focus on the families, which play an important role in the treatment-seeking process of psychotic patients. To help families detect possible signs of psychosis early and establish adequate help faster, we need more focused research on issues involved in seeking help at the family level. An early intervention program should also focus on how families cope with their prodromal or manifest psychotic members.
